# Adsorption and Quantum Chemical Studies on the Inhibition Potentials of Some Thiosemicarbazides for the Corrosion of Mild Steel in Acidic Medium

**DOI:** 10.3390/ijms11062473

**Published:** 2010-06-15

**Authors:** Eno E. Ebenso, David A. Isabirye, Nnabuk O. Eddy

**Affiliations:** 1 Department of Chemistry, North West University (Mafikeng Campus), Private Bag X2046, Mmabatho 2735, South Africa; 2 Department of Chemistry, Ahmadu Bello University, Zaria, Nigeria

**Keywords:** thiosemicarbazides, corrosion inhibitors, quantum chemical calculations, QSAR, adsorption, Fukui function

## Abstract

Three thiosemicarbazides, namely 2-(2-aminophenyl)-N phenylhydrazinecarbothioamide (AP4PT), N,2-diphenylhydrazinecarbothioamide (D4PT) and 2-(2-hydroxyphenyl)-N-phenyl hydrazinecarbothioamide (HP4PT), were investigated as corrosion inhibitors for mild steel in H_2_SO_4_ solution using gravimetric and gasometric methods. The results revealed that they all inhibit corrosion and their % inhibition efficiencies (%IE) follow the order: AP4PT > HP4PT > D4PT. The %IE obtained from the gravimetric and gasometric experiments were in good agreement. The thermodynamic parameters obtained support a physical adsorption mechanism and the adsorption followed the Langmuir adsorption isotherm. Some quantum chemical parameters were calculated using different methods and correlated with the experimental %IE. Quantitative structure activity relationship (QSAR) approach was used on a composite index of some quantum chemical parameters to characterize the inhibition performance of the studied molecules. The results showed that the %IE were closely related to some of the quantum chemical parameters, but with varying degrees. The calculated/theoretical %IE of the molecules were found to be close to their experimental %IE. The local reactivity has been studied through the Fukui and condensed softness indices in order to predict both the reactive centers and to know the possible sites of nucleophilic and electrophilic attacks.

## 1. Introduction

There has been a growing interest in the use of organic compounds as inhibitors for the aqueous corrosion of metals. The protection of metal surfaces against corrosion is an important industrial and scientific topic. Inhibitors are one of the practical means of preventing corrosion, particularly in acidic media. Inhibitors can adhere to a metal surface to form a protective barrier against corrosive agents in contact with metal. The effectiveness of an inhibitor to provide corrosion protection depends to a large extent on the interaction between the inhibitor and the metal surface. The adsorbed inhibitors can affect the corrosion reaction, either by the blocking effect of the adsorbed inhibitor on the metal surface or by the effects attributed to the change in the activation barriers of the anodic and cathodic reactions of the corrosion process. Organic compounds, which can donate electrons to unoccupied d orbitals of metal surface to form coordinate covalent bonds and can also accept free electrons from the metal surface by using their antibonding orbitals to form feedback bonds, constitute excellent corrosion inhibitors. The most effective inhibitors are those compounds containing heteroatoms like nitrogen, oxygen, sulfur and phosphorus, as well as aromatic rings. The inhibitory activity of these molecules is accompanied by their adsorption to the metal surface. Free electron pairs on heteroatoms or *π* electrons are readily available for sharing to form a bond and act as nucleophile centers of inhibitor molecules and greatly facilitate the adsorption process over the metal surface, whose atoms act as electrophiles. Recently, the effectiveness of an inhibitor molecule has been related to its spatial as well as electronic structure [1–4]. Quantum chemical methods are ideal tools for investigating these parameters and are able to provide insight into the inhibitor–surface interaction.

Thiosemicarbazides, thiosemicarbazones and their derivatives have continued to be the subject of extensive investigation in chemistry and biology owing to their broad spectrum of antitumor [5], antibacterial [6,7], antiviral [8–10], antifungal [11], antimalarial [12] and antineoplastic [13] activities, and recently reported corrosion inhibiting properties [14–23]. Recently, Kandemirli and Sagdinc [24] reported on the theoretical studies of corrosion inhibition of some amides and thiosemicarbazones using some quantum chemical calculations. The data available so far are largely incomplete and it is not yet possible to draw very good conclusions about the characteristics of this set of compounds and their derivatives. Therefore, the objective of this study is to present an experimental and theoretical study on the adsorption, electronic and molecular structures of three thiosemicarbazides, namely 2-(2-aminophenyl)-N-phenylhydrazinecarbothioamide (AP4PT), N,2-diphenylhydrazinecarbothioamide (D4PT) and 2-(2-hydroxyphenyl)-*N*-phenyl hydrazinecarbothioamide (HP4PT), used as inhibitors, and to determine the relationship between some quantum chemical parameters/descriptors from the structure of the compounds and the inhibition efficiencies obtained using different methods. Our aim is to also find good theoretical parameters to characterize the inhibition property of the inhibitors, to establish correlations between inhibition efficiencies and some of the electronic properties of the studied molecules using different quantum chemical/theoretical methods, quantitative structure activity relationship (QSAR) approach and local reactivity indices.

## 2. Results and Discussion

### 2.1. Effect of AP4PT, HP4PT and D4PT and Temperature

**Figure 1.**
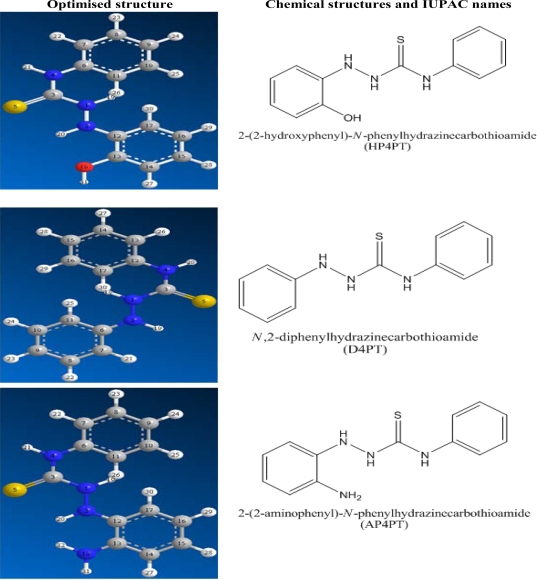
Chemical and optimized structures of the studied thiosemicarbazides.

Figure 1 shows the chemical and optimized structures of the inhibitors (AP4PT, HP4PT and D4PT). Table 1 shows the corrosion rates and the % inhibition efficiencies of AP4PT, HP4PT and D4PT in acid media. The corrosion rate of mild steel for the blank solution (1 M H_2_SO_4_) is higher than those obtained for solutions containing various concentrations of AP4PT, HP4PT and D4PT. This indicates that the corrosion of mild steel in H_2_SO_4_ solution is inhibited by various concentrations of AP4PT, HP4PT and D4PT. It was also found that the corrosion rate of mild steel decreases with increase in the concentration of the inhibitor, but decreases with increasing temperature, which indicates that the inhibitory potentials of AP4PT, HP4PT and D4PT for mild steel corrosion increase with increasing concentration but decrease with increase in temperature. The values of % inhibition efficiencies obtained from the hydrogen evolution method are close to that obtained using the weight loss method (Table 1). From the calculated values of the inhibition efficiencies of AP4PT, HP4PT and D4PT, it is indicative that these inhibitors are adsorption inhibitors and that their inhibition efficiencies decrease in the following trend, AP4PT > HP4PT > D4PT. Furthermore, from the observed trend for the variation of inhibition efficiency with temperature, it is evident that the mechanism of adsorption of the inhibitors on mild steel surface is by a physical adsorption mechanism. For physical adsorption, the inhibition efficiency is expected to decrease with increasing temperature, but for chemical adsorption, the inhibition efficiency is expected to increase with increasing temperature [25].

The activation energies for the corrosion of mild steel in the absence and presence of the inhibitors were calculated using the logarithmic form of the Arrhenius Equation shown below [25]:

(1) $$\text{log}\frac{CR_{2}}{CR_{1}}=\frac{E_{a}}{2.303R}\left(\frac{1}{T_{1}}-\frac{1}{T_{2}}\right)$$

where *CR*_1_ and *CR*_2_ are the corrosion rates of mild steel at the temperatures *T*_1_ (303 K) and *T*_2_ (333 K), respectively. *E_a_* is the activation energy for the reaction and *R* is the molar gas constant. Values of the *E_a_* calculated from Equation 1 are presented in Table 2. The activation energies obtained for the inhibited corrosion of mild steel are within the limit expected (<80 KJ/mol) for the mechanism of physical adsorption, hence the adsorption of AP4PT, HP4PT and D4PT on mild steel surface is consistent with the mechanism of charge transfer from the inhibitor to the metal surface [26].

### 2.2. Thermodynamics/Adsorption Considerations

The heats of adsorption of AP4PT, HP4PT and D4PT on mild steel surface were calculated using the following Equation [25]:

(2) $$Q_{ads}=2.303R\left[\text{log}\left(\frac{\theta_{2}}{1-\theta_{2}}\right)-\text{log}\left(\frac{\theta_{1}}{1-\theta_{1}}\right)\right]x\left(\frac{T_{1X}T_{2}}{T_{2}-T_{1}}\right)kJmol^{-1}$$

where *Q_ads_* is the heat of adsorption, *R* is the gas constant, *θ*_1_ and *θ*_2_ are the degrees of surface coverage of the inhibitors at the temperatures *T*_1_ (303 K) and *T*_2_ (333 K), respectively. Calculated values of *Q_ads_* are negative, indicating that the adsorption of the inhibitors on the mild steel surface is exothermic (see Table 2).

The adsorption characteristics of the inhibitors were investigated by fitting the experimental data obtained for the degrees of surface coverage into different adsorption isotherms. The tests revealed that the adsorption of AP4PT, HP4PT and D4PT can best be described by the Langmuir adsorption isotherm. The Equation for the Langmuir adsorption isotherm can be written as follows [27,28]:

(3) θ= KC × 1/1 + KC)

where K designates the adsorption equilibrium constant and C is the concentration of the inhibitor in the bulk electrolyte. From the rearrangement of Equation 3, Equations 4 and 5 are obtained.

(4) 1/K + C = C/θ

(5) log(C/θ) = logC−logK

**Figure 2.**
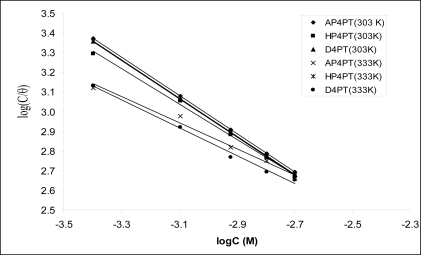
Langmuir isotherm for the adsorption of AP4PT, HP4PT and D4PT on mild steel surface.

Figure 2 shows the plots of values of log(*C*/*θ*) *versus* log*C*. The plots were found to be linear indicating the application of the Langmuir isotherm to the adsorption of AP4PT, HP4PT and D4PT on mild steel surface. Values of adsorption parameters deduced from the Langmuir adsorption isotherms are presented in Table 3. The results obtained indicated that the slopes and *R*^2^ values were very close to unity, which signifies strong adherence of AP4PT, HP4PT and D4PT to the adsorption of the Langmuir model.

The values of the adsorption equilibrium constant (*K*) obtained from the intercept of the Langmuir adsorption isotherms are related to the free energy of adsorption according to Equation 6 [28,29];

(6) $$\Delta G_{ads}^{0}=-2.303RT\;\text{log}(55.5K)$$

where Δ*G*^0^*_ads_* is the free energy of adsorption, R is the gas constant and T is the temperature of the system. Calculated values of the free energies are also presented in Table 3. The free energies ranged from −10.51 to −16.76 kJ/mol and are within the range expected for the transfer of charge from the inhibitor to the metal surface. Therefore, the adsorption of AP4PT, HP4PT and D4PT is spontaneous. Generally, values of Δ*G*^0^*_ads_* up to −20 kJ/mol signify physisorption, the inhibition acts due to electrostatic interactions between the charged molecules and the charged metal, while values around −40 kJ/mol or less are associated with chemisorption as a result of sharing or transfer of electrons from the organic molecules to the metal surface to form a coordinate type of bond (chemisorption). The values obtained from this study ranged from −10.51 to −16.76 kJ/mol, which support the mechanism of physical adsorption [30].

### 2.3. Quantum Chemical Studies

Quantum chemical calculations have been widely used to study reaction mechanisms [31]. They have also been proved to be a very powerful tool for studying inhibition of the corrosion of metals [32–34]. It has been found that the effectiveness of a corrosion inhibitor can be related to its electronic and spatial molecular structure [35–39]. In this study, the relationship between quantum chemical parameters and inhibition efficiency was investigated.

Table 4 shows the values of some quantum chemical parameters, namely the energy of the highest occupied molecular orbital (*E*_HOMO_), energy of the lowest unoccupied molecular orbital (*E*_LUMO_), the energy gap (*E*_LUMO-HOMO_), the total electronic energy of the molecules (*EE*), core core repulsion (CC), dipole moment (*μ*), log*P* (substituent constant - measure of the differential solubility of a compound in two solvents and characterizes the hydrophobicity/hydrophilicity of a molecule), molecular polarizability (pol), cosmo volume (molecular volume) (cosVol) and cosmo area (molecular surface area or solvent accessible molecular surface area) (cosAr). The quantum chemical parameters were computed for five different Hamiltonians, namely, parametric method 6 (PM6), parametric method 3 (PM3), Austin model 1 (AM1), Recife model 1 (RM1) and modified neglect of diatomic overlap (MNDO) [40]. The results obtained from semi empirical computations, are presented in Table 4. As can be seen from Table 4, the *E*_HOMO_, *E*_LUMO_, Δ*E* and dipole moment values calculated for AP4PT by PM3 method heavily deviate from the data obtained from other Hamiltonians. This can be explained as follows. All the semi empirical methods contain sets of parameters. Atomic and diatomic parameters exist in PM6, while MNDO, AM1, PM3, and MNDO-*d* use only single-atom parameters. Not all parameters are optimized for all methods; for example, in MNDO and AM1 the two electron one center integrals are normally taken from atomic spectra. Therefore, in AP4PT, atomic and diatomic parameters are very significant. The frontier molecular orbital energies (*i.e.*, *E*_HOMO_ and *E*_LUMO_) are significant parameters for the prediction of the reactivity of a chemical species. The *E*_HOMO_ is often associated with the electron donating ability of a molecule [36–38]. Therefore, increasing values of *E*_HOMO_ indicates higher tendency for the donation of electron(s) to the appropriate acceptor molecule with low energy and empty molecular orbital. According to Eddy and Ebenso [40], increasing values of *E*_HOMO_ facilitate the adsorption of the inhibitor. Consequently, the inhibition efficiency of the inhibitor would be enhanced by improving the transport process through the adsorbed layer. From Table 4, it is evident that the *E*_HOMO_ for the inhibitors decreases in the order; (AP4PT > HP4PT > D4PT), which is consistent with the experimental % inhibition efficiency results. However, the *E*_LUMO_ decreases in a similar order. This can be explained as follows. The *E*_LUMO_ indicates the ability of the molecule to accept electrons. Therefore, the lower the value of *E*_LUMO_ the more apparent it is that the molecule would accept electrons. Also, the *E*_LUMO-HOMO_ (energy gap) was also found to decrease in the order similar to that of the *E*_LUMO_. Literature reveals that a larger value of the energy gap indicates low reactivity to a chemical species because the energy gap is related to the softness or hardness of a molecule. A soft molecule is more reactive than a hard molecule because a hard molecule has a larger energy gap [37,38].

Values of log*P* (substituent constant) were also found to have a good relationship with the corrosion inhibition efficiencies of the studied inhibitors. Substituent constants are empirical quantities, which account for the variation of the structure and do not depend on the parent structure but vary with the substituent [39]. According to Eddy and Ebenso [40], log*P* accounts for the hydrophobicity of an actual molecule. Hydrophobicity of an organic molecule increases with decreasing water solubility. In corrosion studies, hydrophobicity is related to the mechanism of formation of the oxide/hydroxide layer on the metal surface (which reduces the corrosion process drastically). From the results obtained, based on the increasing value of log*P*, the inhibition efficiencies of the studied thiosemicarbazides increase in the following order, AP4PT > HP4PT > D4PT, which is consistent with experimental obtained % inhibition efficiency results.

The dipole moment (*μ*) is an index that can also be used for the prediction of the direction of a corrosion inhibition process. Dipole moment is the measure of polarity in a bond and is related to the distribution of electrons in a molecule [41]. Although literature is inconsistent on the use of ‘*μ*’ as a predictor for the direction of a corrosion inhibition reaction, it is generally agreed that the adsorption of polar compounds possessing high dipole moments on the metal surface should lead to better inhibition efficiency. Comparison of the results obtained from quantum chemical calculations with experimental inhibition efficiencies indicated that the % inhibition efficiencies of the inhibitors increase with increasing value of the dipole moment.

El Ashry *et al*. [42] noted that core core repulsion energy is a quantum chemical parameter that may have excellent correlation with inhibition efficiency. They reported that the inhibition efficiency of some Schiff bases decreased with increasing value of core core repulsion energy. Similarly, the inhibition efficiencies of the studied thiosemicarbazides were found to decrease with increasing values of core core repulsion. This study on quantum chemical descriptors has been extended to include the total and the electronic energies of the molecules. From the results, it is evident that based on the decreasing values of the total energy (TE) and electronic energy (EE), as well as decreasing value of cosmo area and cosmo volume, the trend for the variation of the inhibition efficiency follows the order similar to experimental %inhibition efficiencies (AP4PT > HP4PT > D4PT).

Polarizability is the ratio of induced dipole moment to the intensity of the electric field. The induced dipole moment is proportional to polarizability [43]. Some attempts have been made to relate the polarizability of some corrosion inhibitors to their inhibition efficiency. According to Arslan *et al*. [38], the minimum polarizability principle (MPP) expects that the natural direction of evolution of any system is towards a state of minimum polarizability. From the results obtained from quantum chemical calculations, the trend for the increase in the inhibition efficiencies of the inhibitors with respect to increasing polarizability correlates well with the order of the experimental % inhibition efficiencies results (AP4PT > HP4PT > D4PT).

**Figure 3.**
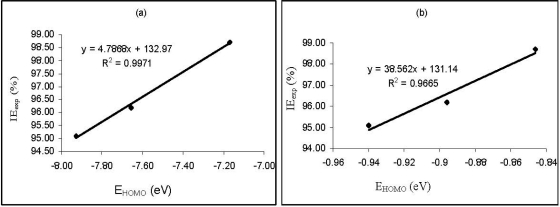

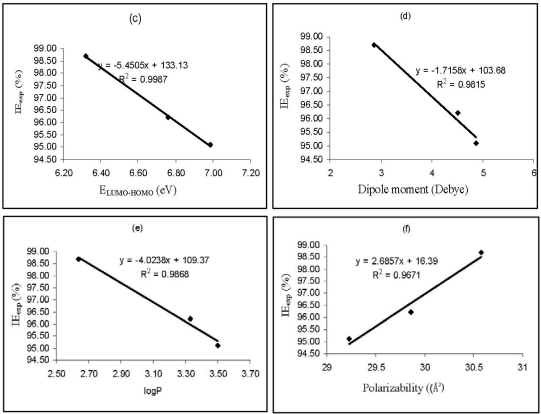
Variation of experimental inhibition efficiency (IEexp) of the studied thiosemicarbazides with **(a)** *E*_HOMO_ **(b)** *E*_LUMO_ **(c)** *E*_LUMO-HOMO_ **(d)** Dipole moment; **(e)** log*P* and **(f)** Polarizability obtained from PM6 calculations.

Correlations between the calculated quantum chemical parameters were also carried out. Figure 3 shows plots for the variation of the experimental inhibition efficiencies with some quantum chemical parameters. The figure reveals that the degree of linearity (*R*^2^) between the plotted quantum chemical parameters and the experimental inhibition efficiencies were very close to unity, which indicated a high degree of linearity. However, the plots were developed from parameters obtained from PM6 Hamiltonians. *R*^2^ values for other Hamiltonians are presented in Table 5. From the results obtained, the highest degree of linearity between the experimental inhibition efficiencies and the *E*_HOMO_, *E*_LUMO_, *E*_LUMO-HOMO_ and μ were obtained from the AM1, PM6, AM1 and MNDO Hamiltonians, respectively. However, *R*^2^ values with respect to *EE*, CC, cosVol and cosAr were relatively low.

### 2.4. Quantitative Structure Activity Relationship (QSAR)

According to Karelson and Lobanov [44], quantitative structure-activity and structure property relationship studies are unquestionably of great importance in modern chemistry and biochemistry. The concept of QSAR/QSPR is to transform searches for compounds with desired properties using chemical intuition and experience into mathematically quantified and computed form. Once a correlation between structure and activity/property is found, any number of compounds, including those not yet synthesized, can readily be screened on the computer [45,46].

Most recent studies on the use of QSPR/QSAR for corrosion employ quantum chemical calculations as an attractive source of new molecular descriptor. According to Vera *et al*. [47], QSAR/QSPR can be used to relate the inhibition efficiency of most inhibitors to structural parameters (quantum and topological), which can be theoretically calculated with the ultimate aim of obtaining a molecular design of new corrosion inhibitors. El Ashry *et al.* [48] also stated that although the QSAR is a useful tool for the development of new corrosion inhibitors, the development of Equations for calculating the corrosion inhibition efficiency may lead to a prediction of the efficiency of some inhibitors.

Attempts were made to establish the relationship between corrosion inhibition efficiencies and the calculated quantum chemical parameters using linear regression analysis. The linear model approximated the inhibition efficiency (*IE*_Theor_) according to the following Equation [49]:

(7) $$IE_{\text{Theor}}=Ax_{i}C_{i}+B$$

where *A* and *B* are the regression coefficients determined by regression analysis, *x_i_* is a quantum chemical index characteristic of the molecule *i*, *C_i_* is the experimental concentration of the inhibitor. Equation 7 did not give a good correlation between the experimental and theoretical inhibition efficiencies therefore, a non linear model, which was first proposed by Lukovits *et al.* [50] for the study of interaction of corrosion inhibitors with metal surface in acidic solutions, was used. This model is based on the Langmuir adsorption isotherm (which assumes that the coverage of the metal surface by the inhibitor’s molecule is the primary cause of corrosion inhibition) and can be written as follows [38]:

(8) $$IE_{\text{Theor}}(\%)=\frac{(Ax_{j}+B)C_{i}}{1+(Ax_{j}+B)C_{i}}\times \;100$$

Using the non linear model, multiple regressions were performed between the inhibition efficiencies of the inhibitors and some quantum chemical parameters/descriptors. The solutions of the above non linear Equation are given by Equations 9 to 13 for PM6, PM3, AM1, RM1 and MNDO, respectively. The corresponding correlation coefficients (r) were 0.821, 0.8589, 0.7500, 0.8155 and 0.8068, respectively. Values of inhibition efficiencies calculated from Equations 9 to 13 are presented in Table 6.

(9) $${\text{IE}}_{\text{Theor}}=\frac{(1.0127{\text{E}}_{\text{HOMO}}+{\text{E}}_{\text{LUMO}}+0.99{\text{E}}_{\text{LUMo}-\text{HOMO}}+\mu +\text{LogP}+\text{Pol}+69.12)*{\text{C}}_{\text{i}}\text{x}\;100}{(1+(1.0127{\text{E}}_{\text{HOMO}}\;+\;{\text{E}}_{\text{LUMO}}\;+0.99{\text{E}}_{\text{LUMo}-\text{HOMO}}+\mu \;+\;\text{LogP}+\text{Pol}+69.12)*{\text{C}}_{\text{i}}}$$

(10) $${\text{IE}}_{\text{Theor}}=\frac{(1.0175{\text{E}}_{\text{HOMO}}+{\text{E}}_{\text{LUMO}}+0.997{\text{E}}_{\text{LUMo}-\text{HOMO}}+\mu +\text{LogP}+\text{Pol}+58.61)*{\text{C}}_{\text{i}}\text{x}\;100}{(1+(1.0175{\text{E}}_{\text{HOMO}}\;+\;{\text{E}}_{\text{LUMO}}\;+0.997{\text{E}}_{\text{LUMo}-\text{HOMO}}+\mu \;+\;\text{LogP}+\text{Pol}+58.61)*{\text{C}}_{\text{i}}}$$

(11) $${\text{IE}}_{\text{Theor}}=\frac{(1.0131{\text{E}}_{\text{HOMO}}+{\text{E}}_{\text{LUMO}}+{\text{E}}_{\text{LUMo}-\text{HOMO}}+0.989\mu +\text{LogP}+\text{Pol}+68.52)*{\text{C}}_{\text{i}}\text{x}\;100}{(1+(1.0131{\text{E}}_{\text{HOMO}}\;+\;{\text{E}}_{\text{LUMO}}\;+{\text{E}}_{\text{LUMo}-\text{HOMO}}+0.989\mu \;+\;\text{LogP}+\text{Pol}+68.52)*{\text{C}}_{\text{i}}}$$

(12) $${\text{IE}}_{\text{Theor}}=\frac{(1.0148{\text{E}}_{\text{HOMO}}+{\text{E}}_{\text{LUMO}}+0.991{\text{E}}_{\text{LUMo}-\text{HOMO}}+\mu +\text{LogP}+\text{Pol}+65.19)*{\text{C}}_{\text{i}}\text{x}\;100}{(1+(1.0148{\text{E}}_{\text{HOMO}}\;+\;{\text{E}}_{\text{LUMO}}\;+0.991{\text{E}}_{\text{LUMo}-\text{HOMO}}+\mu \;+\;\text{LogP}+\text{Pol}+65.19)*{\text{C}}_{\text{i}}}$$

(13) $${\text{IE}}_{\text{Theor}}=\frac{(1.0131{\text{E}}_{\text{HOMO}}+{\text{E}}_{\text{LUMO}}+0.987{\text{E}}_{\text{LUMo}-\text{HOMO}}+\mu +\text{LogP}+\text{Pol}+72.63)*{\text{C}}_{\text{i}}\text{x}\;100}{(1+(1.0131{\text{E}}_{\text{HOMO}}\;+\;{\text{E}}_{\text{LUMO}}\;+0.987{\text{E}}_{\text{LUMo}-\text{HOMO}}+\mu \;+\;\text{LogP}+\text{Pol}+72.63)*{\text{C}}_{\text{i}}}$$

**Figure 4.**
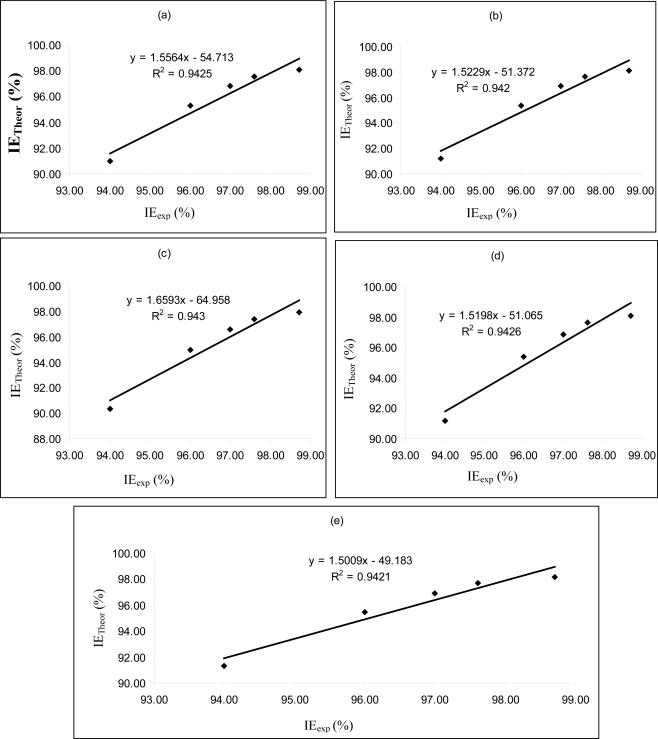
Variation of experimental inhibition efficiency (IE_exp_) of AP4PT with the theoretical inhibition efficiencies (IE_Theor_) calculated for **(a)** PM6 **(b)** PM3 **(c)** AM1 **(d)** RM1 and **(e)** MNDO Hamiltonians.

**Figure 5.**
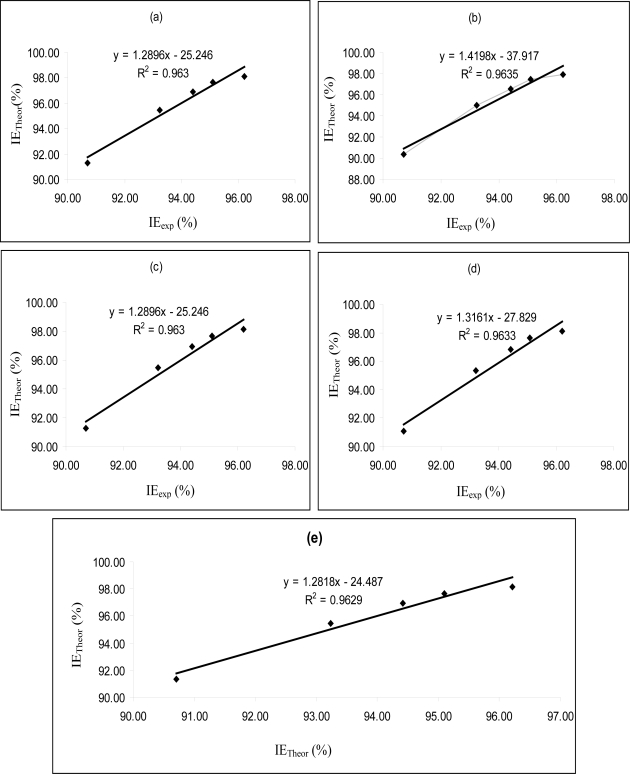
Variation of experimental inhibition efficiency (*IE*_exp_) of HP4PT with the theoretical inhibition efficiencies (*IE*_Theor_) calculated for **(a)** PM6 **(b)** PM3 **(c)** AM1 **(d)** RM1 and **(e)** MNDO Hamiltonians.

**Figure 6.**
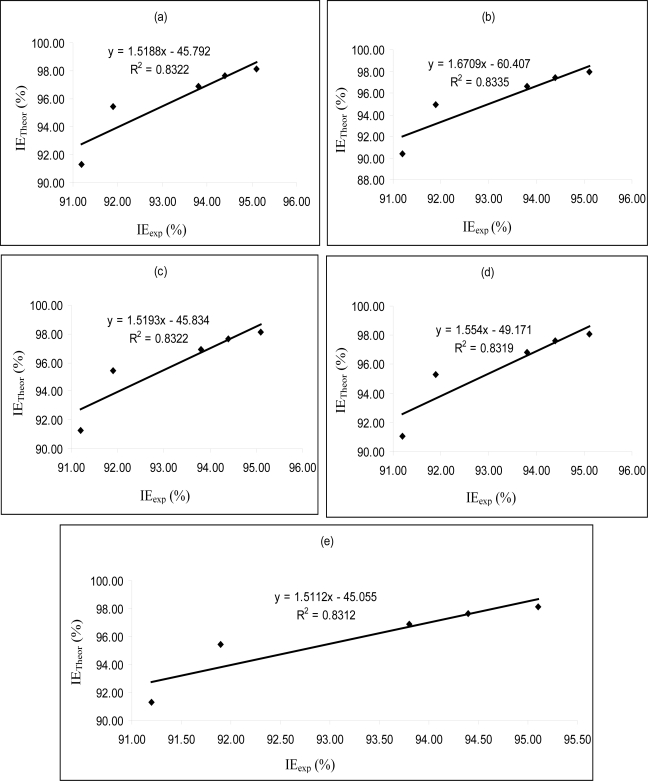
Variation of experimental inhibition efficiency (*IE*_exp_) of D4PT with the theoretical inhibition efficiencies (*IE*_Theor_) calculated for **(a)** PM6 **(b)** PM3 **(c)** AM1 **(d)** RM1 and **(e)** MNDO Hamiltonians.

Figures 4–6 are plots showing the variation of experimental inhibition efficiencies with theoretical inhibition efficiencies for AP4PT, HP4PT and D4PT, respectively. From the plots, it is evident that there is a strong relationship between the theoretical and experimental inhibition efficiencies, indicating that these models can be used to predict the inhibition efficiencies of new corrosion inhibitors that are structurally related to the studied thiosemicarbazides.

### 2.5. Density Functional Theory (DFT)

DFT is based on solving the time independent Schrodinger Equation for the electrons of molecular systems as a function of the positions of the nuclei [51]. The premise behind the density functional theory is that the energy of a molecule can be determined from the electron density instead of a wave function [52]. However, in this study, MP2 method (Moller-Plesset perturbation theory method at level 2) was adopted for computation because of the bulkiness of the studied inhibitors.

#### 2.5.1. Ionization Energy and Electron Affinity

Ionization potential (*IP*) and the electron affinity (*EA*) were calculated using the finite difference approximation as follows [53]:

(14) $$IP=E_{\left(N-1\right)}-E_{\left(N\right)}$$

(15) $$EA=E_{\left(\text{N}\right)}-E_{\left(N+1\right)}$$

where *E*_(_*_N_* _- 1)_, *E*_(_*_N_*_)_ and *E*_(_*_N_* _+ 1)_ are the ground state energies of the system with *N* - 1, *N* and *N* + 1 electrons, respectively. Values of *IE* and *EA* calculated from Equations 14 and 15 are presented in Table 7. The results obtained indicate that the inhibition efficiencies of the inhibitors increase with increasing ionization energy but decrease with decreasing value of electron affinity. This is because IP is directly related with the *E*_HOMO_, while *EA* is related to the *E*_LUMO_. This explains why the trend for the variation of inhibition efficiencies of the inhibitors with IP and EA are similar to those obtained for *E*_HOMO_ and *E*_LUMO_ data.

#### 2.5.2. Global Softness and Hardness

In DFT, the ground state energy *E*(*ρ*) of an atom/molecule can be expressed in terms of its electron density, *ρ*(*r*). The first and second derivatives of *E*(*ρ*) with respect to the number of electrons (*N*) defines the chemical potential (*σ*) and the global hardness (*η*) of a molecule as follows [46]:

(16) $$\sigma ={\left(\delta E/\delta N\right)}_{\nu (r)}$$

(17) $$\eta ={\left(\delta^{2}E/\delta^{2}N\right)}_{\nu (r)}$$

where *v*(*r*) indicates that the differentiation is carried out under constant external potentials. Using the finite difference approximation, the global softness can be evaluated as *S* = 1/(*IP* - *EA*). The global hardness, η, which is the inverse of the global softness can be evaluated using Equation 18 below:

(18) $$S=1/\left[(E_{\left(N-1\right)}-E_{\left(N\right)})-(E_{\left(N\right)}-E_{\left(N+1\right)})\right]$$

Calculated values of S and *η* are also presented in Table 7. From Table 7, it is evident that the inhibitor with the least value of global hardness (hence the highest value of global softness) is the best and *vice versa*. This is because a soft molecule is more reactive than a hard molecule. This observation is consistent with the results obtained from experimental %inhibition efficiencies.

The fraction of electron transferred, *δ*, was calculated using the Equation 19 below [36]:

(19) $$\delta =\left(\chi_{\text{Fe}}-\chi_{\text{inh}}\right)/2(\eta_{\text{Fe}}+\eta_{\text{inh}})$$

where *χ*_Fe_ and *χ*_inh_ are the electronegativity of Fe and the inhibitor, respectively, and can be evaluated as *χ* = (*IP* + *EA*)/2. *η*_Fe_ and *η*_inh_ are the global hardness of Fe and the inhibitor, respectively (calculated using Equation 21). In order to apply Equation 19 to the present study, the theoretical values of *χ*_Fe_ = 7 ev and *η*_Fe_ = 0 were used for the computation of δ values for the various Hamiltonians. Calculated values of δ are presented in Table 7. The results indicate that δ values correlates strongly with experimental inhibition efficiencies. Thus, the highest fraction of electrons is associated with the best inhibitor (AP4PT), while the least fraction is associated with the inhibitor that has the least inhibition efficiency (D4PT).

#### 2.5.3. Local Selectivity

The local selectivity of a corrosion inhibitor is best analyzed using the Fukui function. The Fukui indices permit the distinction of each part of a molecule on the basis of its chemical behavior due to different substituent functional groups.

According to Fuentealba *et al*. [52], the Fukui function can be formally defined as

(20) $$f(r)={\left(\delta \sigma /\delta \nu \right)}_{N}$$

where the functional derivative must be taken at constant number of electron, *N*. If it is assumed that the total energy, *E*, is a function of *v*(*r*) and is an exact differential, then the Maxwell relations between derivatives can be applied to derive the following Equation:

(21) $$f(r)={\left(\delta \rho (r)/\delta N\right)}_{\nu }$$

Equation 21 is the most standard presentation of the Fukui function. The Fukui function is provoked by the fact that if an electron δ is transferred to an *N* electron molecule, it will tend to distribute so as to minimize the energy of the resulting *N* + *δ* electron system. The resulting change in electron density is the nucleophilic (*f*^+^) and electrophilic (*f^−^*) Fukui functions, which can be calculated using the finite difference approximation as follows [52]:

(22) $$f^{+}={\left(\delta \rho (r)/\delta N\right)}_{\upsilon }^{+}=q_{\left(N+1\right)}-q_{\left(N\right)}$$

(23) $$f^{-}={\left(\delta \rho (r)/\delta N\right)}_{\upsilon }^{-}=q_{\left(N\right)}-q_{\left(N-1\right)}$$

where *ρ*, *q*_(_*_N_* _+ 1),_ q_(N)_ and q_(N - 1)_ are the density of electron and the Mulliken charge of the atom with *N* + 1, *N* and *N*-1 electrons.

Calculated values of *q*_(_*_N_* _+ 1),_ *q*_(_*_N_*_)_, *q*_(_*_N_* _- 1),_ *f*^+^ and *f^−^* for AP4PT, HP4PT and D4PT are presented in Tables 7–10. The site for nucleophilic attack is the site where the value of *f*^+^ is maximum, while the site for electrophilic attack is controlled by the values of *f^−^*. Assuming that the protonated forms of the inhibitors’ molecules have a net positive charge, it can be deduced that the sites for nucleophilic attack are the nitrogen atom (N 4) for the three inhibitors. However, the sites for electrophilic attack are in the carbon atoms (C12, C6 and C6) for AP4PT, HP4PT and D4PT, respectively.

**Figure 7.**
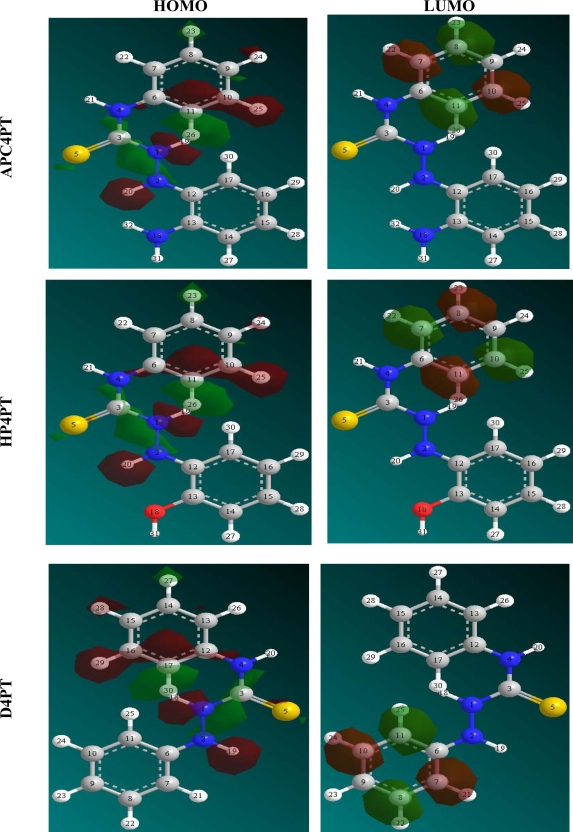
Molecular orbital of the studied thiosemicarbazides showing the HOMO and the LUMO.

The HOMO and LUMO orbitals of AP4PT, HP4PT and D4PT are presented in Figure 7. The figure clearly reveals the information that governs the nucleophilic and electrophilic attacks on the studied inhibitors. The information obtained from the HOMO and LUMO orbitals are consistent with the findings obtained from the Fukui function.

The local softness, *S*, for an atom can be expressed as the product of the condensed Fukui function (*f*) and the global softness (*S*), as follows [52–54];

(24) $$S^{+}=(f^{+})S$$

(25) $$S^{-}=(f^{-})S$$

The local softness contains information similar to those obtained from the condensed Fukui function plus additional information about the total molecular softness, which is related to the global reactivity with respect to a reaction partner. Calculated values of *S*^+^ and *S*^−^ are presented in Tables 8–10. From the results obtained, the sites for electrophilic and nucleophilic attack in the studied thiosemicarbazides are slightly similar. Other indices that can be used to predict the reactive sites of a corrosion inhibitor are the relative nucleophilicity and electrophilicity, which is defined as (*S*^+^/*S*^−^) and (*S*^−^/*S*^+^), respectively. These functions have been successfully applied for the prediction of reactivity sequences of carbonyl compounds toward nucleophilic attack. It was observed that the atoms with highest value of relative nucleophilicity and electrophilicity are similar to those obtained for the Fukui and global softness functions (though the results are not presented here).

## 3. Experimental Techniques

### 3.1. Materials

Materials used for the study were mild steel sheets of composition (wt %); Mn (0.6), P (0.36), C (0.15) and Si (0.03) and the rest Fe. Each sheet was mechanically pressed cut to form different coupons, each of dimension 5 × 4 × 0.11 cm. Each coupon was degreased by washing with ethanol, dipped in acetone and allowed to air dry before they were preserved in a desiccator. All reagents used for the study were Analar grade and double distilled water was used for their preparation. The inhibitors 2-(2-aminophenyl) Nphenylhydrazinecarbothioamide(AP4PT),N,2diphenylhydrazinecarbothioamide(D4PT) and 2-(2-hydroxyphenyl)- phenylhydrazinecarbothioamide(HP4PT) were synthesized as described earlier by Kittur and Mahajan Shetti [55]. The concentrations of inhibitor used for the study was 4 × 10^−4^ to 20 × 10^−4^ M in 1L solution of 1M H_2_SO_4_.

### 3.2. Gravimetric (Weight Loss) Method

In the gravimetric experiment, a previously weighed mild steel coupon was completely immersed in 250 mL of the test solution in an open beaker. The beaker was inserted into a water bath maintained at 303 K. After every 24 h, each sample was withdrawn from the test solution, washed in a solution containing 50% NaOH and 100 g/L of zinc dust. The washed coupons were dipped in acetone and allowed to air dry before re-weighing. The difference in weight for a period of 168 h (7 days) was taken as total weight loss. The experiments were repeated at 333 K. From the weight loss results, the inhibition efficiency (%I) of the inhibitor, degree of surface coverage and corrosion rates were calculated using Equations 26, 27, and 28. respectively [56];

(26) $$\%\text{IE}=(1-W_{1}/W_{2})\times 100$$

(27) $$\theta =1-W_{1}/W_{2}$$

(28) $$CR({\text{gh}}^{-1}{\text{cm}}^{-2})=W/At$$

where *W*_1_ and *W*_2_ are the weight losses (g) for mild steel in the presence and absence of the inhibitor in H_2_SO_4_ solution, *θ* is the degree of surface coverage of the inhibitor, *A* is the area of the mild steel coupon (in cm^2^), t is the period of immersion (in hours) and *W* is the weight loss of mild steel after time, *t*. All the measurements were performed in triplicate and the mean value recorded.

### 3.3. Gasometric (Hydrogen Evolution) Method

The hydrogen evolution technique (gasometric) experiment was carried out at 303 K as described in literature [56]. From the volume of hydrogen evolved per minute, inhibition efficiencies were calculated using Equation 29 below.

(29) $$\%\text{IE}=\left(1-\frac{V_{Ht}^{1}}{V_{Ht}^{o}}\right)x100$$

where $V_{Ht}^{1}$ and $V_{Ht}^{o}$ are the volumes of H_2_ gas evolved at time ‘*t*’ for inhibited and uninhibited solutions, respectively.

### 3.4. Quantum Chemical Calculations

Single point energy calculations were carried out using AM1, PM6, PM3, MNDO and RM1 Hamiltonian in the MOPAC 2008 software for Windows [57]. Calculations were performed on an IBM compatible Intel Pentium IV (2.8 GHz, 4 GB RAM) computer. The following quantum chemical parameters were calculated: the energy of the highest occupied molecular orbital (*E*_HOMO_), the energy of the lowest unoccupied molecular orbital (*E*_LUMO_), the dipole moment (*μ*), the total energy (*TE*), the electronic energy (*EE*), the ionization potential, the cosmo area (cosAr) and the cosmo volume (cosVol). The polarizability (Pol) and log*P* were also calculated using Hyperchem release 8.0.3 for windows [58]. The Mulliken and Lowdin charges (*q*) for nucleophilic and electrophilic attacks were computed using GAMES computational software [59]. The correlation type and method used for the calculation was MP2 while the basis set was set to STO3G*.

Statistical analyses were performed using SPSS program version 15.0 for Windows. Non-linear regression analyses were performed by unconstrained sum of squared residuals for loss function and estimation methods of Levenberg-Marquardt using SPSS program version 15.0 for Windows [60].

## 4. Conclusions

All the methods used showed that the three thiosemicarbazides possess good inhibition properties for the corrosion of mild steel in H_2_SO_4_ at the temperatures studied and their % inhibition efficiencies increased with increasing concentration of the inhibitors and decreasing temperature. The % inhibition efficiencies obtained from the gravimetric and gasometric experiments were in good agreement. The thermodynamic parameters obtained support a physical adsorption mechanism. Adsorption of the inhibitors on the mild steel surface followed the Langmuir adsorption isotherm. The calculated/theoretical % inhibition efficiencies of the molecules were found to be close to their experimental % inhibition efficiencies. From the local reactivity indices, it was found that the sites for electrophilic attack are in the carbon atoms (C12, C6 and C6) for AP4PT, HP4PT and D4PT respectively and that for nucleophilic attack are the nitrogen atom (N 4) for the three inhibitors.

## Figures and Tables

**Table 1.** Inhibition efficiencies (%IE) and corrosion rates (CR) of the studied thiosemicarbazides for the corrosion of mild steel in H_2_SO_4_ solutions using both the weight loss (at 303 and 333 K) and hydrogen evolution techniques at 303 K only.

**Systems**	**%IE (CR)**	
	**303 K**	**333 K**
1 M H_2_SO_4_ (Blank)	(34.69)	(96.23)
4 × 10^−4^ M AP4PT + 1 M H_2_SO_4_	94.0 a(2.08) [92.2]^b^	78.92 (20.29)
8 × 10^−4^ M AP4PT + 1 M H_2_SO_4_	96.0 (1.37) [94.6]	90.80 (8.85)
12 × 10^−4^ M AP4PT + 1 M H_2_SO_4_	97.0(1.04) [95.9]	91.90 (7.79)
16 × 10^−4^ M AP4PT + 1 M H_2_SO_4_	97.6 (0.82) [96.1]	92.80 (6.93)
20 × 10^−4^ M AP4PT + 1 M H_2_SO_4_	98.7 (0.44) [97.2]	93.40 (6.35)
4 × 10^−4^ M HP4PT + 1 M H_2_SO_4_	90.7 (3.23) [88.6]	52.80 (45.42)
8 × 10^−4^ M HP4PT + 1 M H_2_SO_4_	93.1 (2.38) [91.8]	75.90 (23.19)
12 × 10^−4^ M HP4PT + 1 M H_2_SO_4_	94.4 (1.93) [92.9]	79.00 (20.21)
16 × 10^−4^ M HP4PT + 1 M H_2_SO_4_	95.1 (1.70) [94.2]	89.84 (9.78)
20 × 10^−4^ M HP4PT + 1 M H_2_SO_4_	96.2 (1.30) [95.0]	91.21 (8.46)
4 × 10^−4^ M D44PT + 1 M H_2_SO_4_	91.2 (3.05) [90.1]	54.42 (43.86)
8 × 10^−4^ M D4PT + 1 M H_2_SO_4_	91.9 (2.79) [88.4]	66.40 (32.33)
12 × 10^−4^ M D4PT + 1 M H_2_SO_4_	93.8 (2.15) [91.5]	70.50 (28.39)
16 × 10^−4^ M D4PT + 1 M H_2_SO_4_	94.4 (1.93) [93.2]	79.20 (20.02)
20 × 10^−4^ M D4PT + 1 M H_2_SO_4_	95.1 (1.70) [93.9]	89.98 (9.64)

a %IE obtained from the weight loss technique.

b %IE obtained from the hydrogen evolution technique.

**Table 2.** Some thermodynamics parameters for the adsorption of the studied thiosemicarbazides using the weight loss technique.

**Systems**	**Activation Energy**, ***E*_*a*_(kJ mol^−1^)**	**Heat of adsorption**, ***Q*_*ads*_(kJ mol^−1^)**
1 M H_2_SO_4_ (Blank)	28.28	-
4 × 10^−4^ M AP4PT + 1 M H_2_SO_4_	63.13	−30.03
8 × 10^−4^ M AP4PT + 1 M H_2_SO_4_	51.72	−18.64
12 × 10^−4^ M AP4PT + 1 M H_2_SO_4_	55.83	−21.97
16 × 10^−4^ M AP4PT + 1 M H_2_SO_4_	59.15	−24.10
20 × 10^−4^ M AP4PT + 1 M H_2_SO_4_	74.00	−35.24
4 × 10^−4^ M HP4PT + 1 M H_2_SO_4_	73.27	−45.42
8 × 10^−4^ M HP4PT + 1 M H_2_SO_4_	63.11	−30.52
12 × 10^−4^ M HP4PT + 1 M H_2_SO_4_	65.10	−31.46
16 × 10^−4^ M HP4PT + 1 M H_2_SO_4_	48.49	−16.49
20 × 10^−4^ M HP4PT + 1 M H_2_SO_4_	51.91	−18.71
4 × 10^−4^ M D44PT + 1 M H_2_SO_4_	73.89	−45.33
8 × 10^−4^ M D4PT + 1 M H_2_SO_4_	67.91	−36.66
12 × 10^−4^ M D4PT + 1 M H_2_SO_4_	71.53	−38.71
16 × 10^−4^ M D4PT + 1 M H_2_SO_4_	64.83	−31.21
20 × 10^−4^ M D4PT + 1 M H_2_SO_4_	48.11	−16.17

**Table 3.** Langmuir parameters for the adsorption for AP4PT, HP4PT and D4PT on mild steel surface.

**Inhibitor**	**Temperature (K)**	**Slope**	**log*K***	***ΔG*^0^(kJ mol^−1^)**	***R*^2^**
**AP4PT**	303	0.971	0.0716	−10.51	1.0000
	333	0.6625	0.8893	−16.76	0.9843
**HP4PT**	303	0.8987	0.9975	−15.88	0.9975
	333	0.9727	0.051	−11.42	0.9999
**D4PT**	303	0.9644	0.0786	−10.55	1.0000
	333	0.7074	0.7243	−15.71	0.9938

**Table 4.** Quantum chemical parameters for the studied thiosemicarbazides.

**Inhibitor**	**Models**	***E*_HOMO_(eV)**	***E*_LUMO_(eV)**	**Δ*E*(eV)**	***EE*(eV)**	**C-C (eV)**	**cosAr (Å^2^)**	**cosVol (Å^3^)**	***μ*(Debye)**	**log*P***	**Pol (Å^3^)**
**AP4PT**	**PM6**	−7.168	−0.846	6.322	−19082.68	16440.61	264.16	283.67	2.874	2.64	30.58
	**PM3**	−4.986	−2.219	2.767	−18634.24	16068.10	264.16	283.67	6.549	2.64	30.58
	**AMI**	−7.390	−0.522	6.868	−19175.65	16316.84	264.16	283.67	3.050	2.64	30.58
	**RMI**	−7.197	−0.450	6.747	−19276.05	16439.86	264.16	283.67	3.473	2.64	30.58
	**MNDO**	−7.441	−0.531	6.910	−19245.14	16350.65	264.16	283.67	3.076	2.64	30.58
**HP4PT**	**PM6**	−7.658	−0.896	6.762	−19094.75	16353.37	263.66	282.72	4.517	3.33	29.86
	**PM3**	−7.769	−1.058	6.711	−18716.62	16028.44	263.66	282.72	4.642	3.33	29.86
	**AMI**	−7.854	−0.538	7.316	−19220.30	16260.58	263.66	282.72	4.550	3.33	29.86
	**RMI**	−7.678	−0.481	7.197	−19304.37	16375.01	263.66	282.72	5.067	3.33	29.86
	**MNDO**	−7.889	−0.546	7.343	−19290.79	16293.87	263.66	282.72	4.460	3.33	29.86
**D4PT**	**PM6**	−7.927	−0.940	6.987	−17084.54	14634.46	254.24	270.42	4.870	3.50	29.23
	**PM3**	−8.014	−1.131	6.883	−16675.34	14280.63	254.24	270.42	5.098	3.50	29.23
	**AMI**	−8.103	−0.598	7.505	−17145.89	14506.42	254.24	270.42	4.854	3.50	29.23
	**RMI**	−7.914	−0.537	7.377	−17240.09	14627.01	254.24	270.42	5.307	3.50	29.23
	**MNDO**	−8.112	−0.605	7.507	−17213.07	14538.32	254.24	270.42	4.806	3.50	29.23

**Table 5.** Correlation coefficients, *r* (degree of linearity, *R*^2^) between quantum chemical parameters and experimental inhibition efficiencies of the studied thiosemicarbazides.

**Quantum parameters**	**PM6**	**PM3**	**AM1**	**RM1**	**MNDO**
***E*_HOMO_(eV)**	0.9916 (0.9971)	0.9917 (0.9481)	0.8800 (0.9977)	0.9362 (0.993)	0.8669 (0.9910)
***E*_LUMO_(eV)**	−0.998 (0.9665)	−0.994 (0.8766)	−0.9983 (0.7326)	−0.9989 (0.8538)	−0.9980 (0.7254)
***E*_LUMO-HOMO_(eV)**	−0.7678 (0.9987)	−0.5319 (0.931)	−0.7552 (0.9999)	−0.7410 (0.9995)	−0.7420 (0.9989)
***EE*(eV)**	0.7977 (0.5357)	0.5777 (0.5053)	0.7847 (0.5221)	0.7733 (0.5290)	0.7730 (0.5217)
**CC (eV)**	0.7989 (0.5834)	0.5982 (0.5602)	0.7953 (0.5681)	0.7816 (0.5722)	0.7838 (0.5683)
**cosAr (Å^2^)**	0.8104 (0.5853)	0.6138 (0.5853)	0.8071 (0.5853)	0.7936 (0.5853)	0.7958 (0.5853)
**cosVol (Å^3^)**	−0.9819 (0.6045)	0.9542 (0.6045)	−0.9814 (0.6045)	−0.9784 (0.6045)	−0.9899 (0.6048)
***μ*(Debye)**	−0.9857 (0.9815)	−0.9934 (0.7412)	−0.9867 (0.9792)	−0.9901 (0.9672)	−0.9896 (0.9874)
**log*P***	0.9918 (0.9868)	0.9162 (0.9868)	0.9910 (0.9668)	0.9878 (0.9668)	0.9884 (0.9868)
**Polarizability (Å^2^)**	0.9985 (0.9671)	0.9737 (0.9671)	0.9989 (0.9671)	0.9997 (0.9671)	0.9960 (0.9671)

**Table 6.** Theoretical inhibition efficiencies of the studied thiosemicarbazides obtained from various models.

**Inhibitor**	**C (M)**	**PM6 (%)**	**PM3 (%)**	**AM1 (%)**	**RM1 (%)**	**MNDO (%)**
**AP4PT**	4 × 10^−4^	90.98	91.18	90.37	91.20	91.31
	8 × 10^−4^	95.27	95.39	94.94	95.40	95.46
	12 × 10^−4^	96.80	96.88	96.57	96.88	96.92
	16 × 10^−4^	97.58	97.64	97.40	97.64	97.68
	20 × 10^−4^	98.05	98.10	97.91	98.11	98.13

**HP4PT**	4 × 10^−4^	91.30	90.40	91.30	91.11	91.35
	8 × 10^−4^	95.45	94.96	95.45	95.34	95.48
	12 × 10^−4^	96.92	96.58	96.92	96.84	96.94
	16 × 10^−4^	97.67	97.41	97.67	97.61	97.68
	20 × 10^−4^	98.13	97.92	98.13	98.08	98.14

**D4PT**	4 × 10^−4^	91.28	90.39	91.28	91.07	91.32
	8 × 10^−4^	95.44	94.95	95.44	95.33	95.47
	12 × 10^−4^	96.91	96.58	96.92	96.83	96.93
	16 × 10^−4^	97.67	97.41	97.67	97.61	97.68
	20 × 10^−4^	98.13	97.92	98.13	98.08	98.14

**Table 7.** Calculated quantum chemical descriptors for the studied thiosemicarbazides.

**Inhibitor**	**Model**	***E*_*N*_(eV)**	***E*_*N*− 1_(eV)**	***E*_*N*+ 1_(eV)**	***IP*(eV)**	***EA*(eV)**	***η*(eV)**	***S*(/eV)**	***χ*(eV)**	***δ***
**AP4PT**	**PM6**	−2642.06	−2635.75	−2643.14	6.31	1.08	5.23	0.19	3.69	0.3160
	**PM3**	−2566.14	−2562.45	−2570.28	5.69	2.14	3.55	0.28	3.92	0.4345
	**AM1**	−2858.81	−2852.21	−2858.91	6.60	0.10	6.50	0.15	3.35	0.2808
	**RM1**	−2836.25	−2829.91	−2837.33	6.34	1.08	5.26	0.19	3.71	0.3127
	**MNDO**	−2894.50	−2887.83	−2895.06	6.67	0.56	6.11	0.16	3.62	0.2770
**HP4PT**	**PM6**	−2741.38	−2734.67	−2743.09	6.71	1.71	5.00	0.20	4.21	0.2790
	**PM3**	−2688.18	−2681.28	−2687.19	6.90	−0.99	7.89	0.13	2.95	0.2563
	**AM1**	−2959.71	−2952.78	−2959.76	6.93	0.05	6.88	0.15	3.49	0.2551
	**RM1**	−2929.36	−2922.64	−2930.42	6.72	1.06	5.66	0.18	3.89	0.2747
	**MNDO**	−2996.92	−2989.95	−2997.48	6.97	0.56	6.41	0.16	3.77	0.2523
**D4PT**	**PM6**	−2450.08	−2443.10	−2451.81	6.98	1.73	5.25	0.19	4.36	0.2519
	**PM3**	−2394.72	−2387.57	−2395.94	7.15	1.22	5.93	0.17	4.18	0.2374
	**AM1**	−2639.48	−2632.31	−2639.63	7.17	0.15	7.02	0.14	3.66	0.2379
	**RM1**	−2613.08	−2606.13	−2614.17	6.95	1.09	5.86	0.17	4.02	0.2543
	**MNDO**	−2674.75	−2667.56	−2675.36	7.19	0.61	6.58	0.15	3.90	0.2356

**Table 8.** Fukui and global softness indices for nucleophilic and electrophilic attacks in HP4PT calculated from Mulliken (Lowdin) charges.

**Atom (No)**	***f*_*x*_^+^(|e|)**	***f*_*x*_^*–*^(|e|)**	***S*_*x*_^+^(eV|e|)**	***S*_*x*_^–^(eV|e|)**
**1 N**	–0.0245(−0.0346)	0.5191(0.3439)	–0.0059(−0.0083)	0.0825(0.1246)
**2 N**	–0.0023(0.0003)	2.7382(2.8610)	–0.0006(0.0001)	0.6866(0.6572)
**3 C**	**0.0009**(0.0042)	–3.5149(−3.1289)	0.0002(0.0010)	–0.7509(−0.8436)
**4 N**	**0.0011(0.0071)**	–5.1867(−4.9549)	0.0003(0.0017)	–1.1892(−1.2448)
**5 S**	–0.1030(−0.0974)	1.6665(1.3058)	–0.0247(−0.0234)	0.3134(0.4000)
**6 C**	–0.0198(−0.0132)	–3.8634(−3.9023)	–0.0048(−0.0032)	–0.9366(−0.9272)
**7 C**	–0.0841(−0.1110)	–4.0753(−4.0663)	–0.0202(−0.0266)	–0.9759(−0.9781)
**8 C**	–0.1022(−0.1425)	–4.0387(−4.0031)	–0.0245(−0.0342)	–0.9607(−0.9693)
**9 C**	0.0000(0.0131)	–4.0731(−4.0514)	0.0000(0.0031)	–0.9723(−0.9775)
**10 C**	–0.0928(−0.1269)	–4.0352(−4.0019)	–0.0223(−0.0305)	–0.9605(−0.9684)
**11 C**	–0.1004(−0.1281)	–4.0622(−4.1127)	–0.0241(−0.0307)	–0.9870(−0.9749)
**12 C**	–0.0070(−0.0075)	**4.1171(4.0717)**	–0.0017(−0.0018)	0.9772(0.9881)
**13 C**	–0.0086(−0.0082)	**4.1127**(4.0689)	–0.0021(−0.0020)	0.9765(0.9870)
**14 C**	–0.0084(−0.0103)	3.9200(3.9422)	–0.0020(−0.0025)	0.9461(0.9408)
**15 C**	–0.0070(−0.0085)	3.9152(3.9360)	–0.0017(−0.0020)	0.9446(0.9396)
**16 C**	–0.0048(−0.0045)	3.9426(3.9744)	–0.0012(−0.0011)	0.9539(0.9462)
**17 C**	0.0012(0.0057)	3.9019(3.9011)	0.0003(0.0014)	0.9363(0.9365)
**18 O**	–0.0010(−0.0014)	1.6743(1.7849)	–0.0002(−0.0003)	0.4284(0.4018)

**Table 9.** Fukui and global softness indices for nucleophilic and electrophilic attacks in D4PT calculated from Mulliken (Lowdin) charges.

**Atom (No)**	***f*_*x*_^+^(|e|)**	***f*_*x*_^−^(|e|)**	***S*_*x*_^+^(eV|e|)**	***S*_*x*_^−^(eV|e|)**
**1 N**	–0.0460(−0.0547)	–0.0527(−0.0617)	–0.0017(−0.0021)	–0.0020(−0.0023)
**2 N**	0.0059(0.0104)	–0.0063(−0.0101)	0.0002(0.0004)	–0.0002(−0.0004)
**3 C**	–0.1280(−0.1782)	**0.0106(0.0241)**	–0.0049(−0.0068)	0.0004(0.0009)
**4 N**	**0.0241(0.0366)**	–0.0263(−0.0361)	0.0009(0.0014)	–0.0010(−0.0014)
**5 S**	–0.2715(−0.2551)	–0.5881(−0.6199)	–0.0103(−0.0097)	–0.0223(−0.0236)
**6 C**	–0.0031(−0.0016)	**0.0143(0.0227)**	–0.0001(−0.0001)	0.0005(0.0009)
**7 C**	–0.0158(−0.0174)	–0.0167(−0.0201)	–0.0006(−0.0007)	–0.0006(−0.0008)
**8 C**	–0.0097(−0.0110)	–0.0057(−0.0049)	–0.0004(−0.0004)	–0.0002(−0.0002)
**9 C**	–0.0213(−0.0275)	–0.0245(−0.0317)	–0.0008(−0.0010)	–0.0009(−0.0012)
**10 C**	–0.0070(−0.0059)	–0.0048(−0.0030)	–0.0003(−0.0002)	–0.0002(−0.0001)
**11 C**	–0.0069(−0.0075)	–0.0151(−0.0183)	–0.0003(−0.0003)	–0.0006(−0.0007)
**12 C**	–0.0653(−0.0862)	**0.0082(0.0217)**	–0.0025(−0.0033)	0.0003(0.0008)
**13 C**	–0.0207(−0.0232)	–0.0251(−0.0298)	–0.0008(−0.0009)	–0.0010(−0.0011)
**14 C**	–0.0256(−0.0293)	–0.0052(−0.0026)	–0.0010(−0.0011)	–0.0002(−0.0001)
**15 C**	–0.0359(−0.0483)	–0.0268(−0.0342)	–0.0014(−0.0018)	–0.0010(−0.0013)
**16 C**	–0.0263(−0.0291)	–0.0047(0.0006)	–0.0010(−0.0011)	–0.0002(0.0000)
**17 C**	–0.0251(−0.0269)	–0.0456(−0.0579)	–0.0010(−0.0010)	–0.0017(−0.0022)

**Table 10.** Fukui and global softness indices for nucleophilic and electrophilic attacks in AP4PT calculated from Mulliken (Lowdin) charges.

**Atom (No)**	***f*_*x*_^+^(|e|)**	***f*_*x*_^−^(|e|)**	***S*_*x*_^+^(eV|e|)**	***S*_*x*_**^−^**(eV|e|)**
**1 N**	–0.0239(−0.0340)	–0.0113(−0.0051)	–0.0045(−0.0065)	–0.0022(−0.0010)
**2 N**	–0.0034(−0.0010)	–0.1666(−0.2265)	–0.0007(−0.0002)	–0.0317(−0.0430)
**3 C**	**0.0011(0.0044)**	–0.0009(0.0002)	0.0002(0.0008)	–0.0002(0.0000)
**4 N**	**0.0009(0.0070)**	–0.0148(−0.0176)	0.0002(0.0013)	–0.0028(−0.0033)
**5 S**	–0.1005(−0.0948)	–0.1067(−0.1053)	–0.0191(−0.0180)	–0.0203(−0.0200)
**6 C**	–0.0178(−0.0102)	**0.0055(0.0130)**	–0.0034(−0.0019)	0.00105(0.0025)
**7 C**	–0.0879(−0.1168)	–0.0190(−0.0230)	–0.0167(−0.0222)	–0.0036(−0.0044)
**8 C**	–0.1014(−0.1408)	–0.0069(−0.0064)	–0.0193(−0.0268)	–0.0013(−0.0012)
**9 C**	0.0007(0.0142)	–0.0194(−0.0237)	0.0001(0.00270)	–0.0037(−0.0045)
**10 C**	–0.0947(−0.1304)	–0.0013(0.0032)	–0.0180(−0.0248)	–0.0003(0.0006)
**11 C**	–0.0990(−0.1257)	–0.0272(−0.0362)	–0.0188(−0.0239)	–0.0052(−0.0069)
**12 C**	**0.0020(0.0051)**	–0.0074(−0.0025)	0.0004(0.0010)	–0.0014(−0.0005)
**13 C**	–0.0106(−0.0116)	–0.0261(−0.0257)	–0.0020(−0.0022)	–0.0050(−0.0049)
**14 C**	–0.0075(−0.0088)	–0.0221(−0.0227)	–0.0014(−0.0017)	–0.0042(−0.0043)
**15 C**	–0.0123(−0.0155)	–0.0487(−0.0639)	–0.0023(−0.0030)	–0.0093(−0.0121)
**16 C**	–0.0015(0.0004)	–0.0255(−0.0282)	–0.0003(0.0001)	–0.0049(−0.0054)
**17 C**	–0.0041(−0.0018)	–0.0553(−0.0682)	–0.0008(−0.0003)	–0.0105(−0.0130)
**18 N**	–0.0036(−0.0042)	–0.6434(−0.4673)	–0.0007(−0.0008)	–0.1223(−0.0888)
